# Comparative analysis of anesthetic protocols in spontaneously hypertensive rats with sepsis-induced hemodynamic instability

**DOI:** 10.3389/fvets.2026.1866908

**Published:** 2026-08-19

**Authors:** Maria Alcione Silva Gomes, Aline Aparecida Macedo Marques, Gabriela Pereira da Silva, Luana Ale Bertoncello Pael, Maria Medina de Azevedo, Maria Luiza Fidelis da Silva, Joyner David Anaya Miranda, Bianca Viana Silva, Telma Lélia Gonçalves Schultz de Carvalho, Isabela Marafon Souza Féliz, Sílvia Beatriz Bürger Tinelli, Juliana Cappellari, Salviano Tramontin Bellettini, Emerson Luiz Botelho Lourenço, Arquimedes Gasparotto Junior

**Affiliations:** 1 Laboratory of Cardiovascular Pharmacology (LaFaC), Faculty of Health Sciences, Federal University of Grande Dourados (UFGD), Dourados, Mato Grosso do Sul, Brazil; 2 University Center of Grande Dourados (UNIGRAN), Dourados, Mato Grosso do Sul, Brazil; 3 Graduate Program in Biotechnology Applied to Agriculture, Paranaense University, Umuarama, Paraná, Brazil; 4 Graduate Program in Medicinal Plants and Phytotherapy in Primary Health Care, Paranaense University, Umuarama, Paraná, Brazil; 5 Graduate Program in Animal Science with Emphasis on Bioactive Products, Paranaense University, Umuarama, Paraná, Brazil

**Keywords:** dexmedetomidine, diazepam, fentanyl, isoflurane, ketamine, xylazine

## Abstract

**Introduction:**

Sepsis remains a significant clinical challenge characterized by complex inflammatory responses and hemodynamic instability, often modeled via cecal ligation and puncture (CLP). This study investigated the cardiovascular impacts of various anesthetic protocols in spontaneously hypertensive rats (SHR) subjected to abdominal sepsis.

**Methods:**

Septic SHR were allocated into five anesthetic groups: ketamine-xylazine, fentanyl-diazepam, fentanyl-dexmedetomidine, ketamine-dexmedetomidine, and inhaled isoflurane (2–3%), alongside an unanesthetized septic control. Evaluations included electrocardiography, arterial blood pressure monitoring, blood gas analysis, biochemical profiling, mesenteric vascular reactivity, and histopathology of the heart, kidney, and aorta.

**Results:**

Injectable combinations (ketamine-xylazine, fentanyl-diazepam, fentanyl-dexmedetomidine, and ketamine-dexmedetomidine) induced significant electrocardiographic alterations, hypotension, tissue hypoxia, increased oxidative stress, and elevated serum creatinine. Conversely, isoflurane-anesthetized rats maintained hemodynamic and biochemical stability, showing no significant deviations from the septic control group.

**Conclusion:**

Isoflurane demonstrated superior cardiovascular safety in hypertensive rats during sepsis-induced instability. These findings suggest that while isoflurane is the preferred anesthetic for this specific model, protocol selection must be tailored to the experimental context.

## 1 Introduction

The selection of appropriate anesthetic protocols is a fundamental challenge in preclinical research, as anesthesia acts as a major confounding factor that can significantly alter hemodynamic parameters (1, 2). In invasive cardiovascular studies, chemical restraint is necessary for accurate assessment; however, both inhalant and intravenous anesthetics can precipitate significant fluctuations in blood pressure, potentially masking or exacerbating the physiological responses under investigation (3, 4). These agents induce systemic effects across neuronal, cardiorespiratory, metabolic, and immunological axes, with the magnitude of these alterations being highly dependent on the chosen regimen (5).

This challenge is particularly pronounced in experimental models of sepsis, a condition characterized by profound circulatory instability. Septic shock induces acute cardiovascular failure through systemic vasodilation, triggered by bacterial components such as lipopolysaccharides (LPS). The resulting host immune response—including the release of pro-inflammatory cytokines—directly targets the cardiovascular system, leading to clinical deterioration (6). When combined with the inherent cardiovascular impacts of anesthesia, this instability necessitates meticulous anesthetic selection to ensure both animal welfare and the integrity of hemodynamic data (7, 8).

Despite the importance of identifying agents that minimize cardiovascular disruption, there is a lack of standardized protocols tailored to the septic state. Furthermore, while conventional rats are often used in these models, they may not adequately represent the susceptibility of patients with pre-existing cardiovascular comorbidities to septic complications. The use of spontaneously hypertensive rats (SHR) in this study provides a more clinically relevant model, as these animals possess a baseline of cardiovascular dysfunction that more closely mimics the vulnerability of human populations with hypertension—a major risk factor for adverse outcomes in sepsis (9, 10).

Consequently, the objective of the present study was to identify an anesthetic protocol that minimizes cardiovascular interference in a sepsis model. By evaluating different anesthetic regimens in SHR subjected to abdominal sepsis, we aim to bridge the critical gap in optimizing experimental procedures that require invasive hemodynamic monitoring in the presence of systemic inflammation.

## 2 Materials and methods

### 2.1 Drugs and reagents

The following drugs were used: ketamine hydrochloride (50 mg/mL, 10 mL vial), heparin (5.000 UI/mL, 5 mL vial), fentanyl citrate (0.0785 mg/mL, 10 mL vial), diazepam (5 mg/2 mL, 2 mL ampoule), and isoflurane (100 mL bottle), all from Cristália (Itapira, Brazil); 2% xylazine hydrochloride (Syntec, Santana de Parnaíba, Brazil, 10 mL vial); and dexmedetomidine hydrochloride (União Química, São Paulo, Brazil, 100 μg/mL, 2 mL vial). Acetylcholine, phenylephrine, and sodium nitroprusside were purchased from Sigma-Aldrich (St. Louis, MO, USA) in analytical grade purity (5 g powder vials).

### 2.2 Animals

Sixty male spontaneously hypertensive rats (SHR), aged 3 months and weighing 300–320 g, were obtained from the Central Animal Facility of the Federal University of Grande Dourados (UFGD). The animals were housed at the FCS/UFGD research vivarium under controlled conditions, with a temperature range of 22–25 °C and a 12-h light/dark cycle. The rats were kept in groups of 3–4 per cage (49 × 34 × 16 cm), using wood shavings as bedding, with free access to food and water ad libitum. All procedures were previously approved by the Ethics Committee on Animal Use of UFGD (Protocol No. 23021) and were conducted in accordance with the Brazilian Guidelines for the Care and Use of Animals for Scientific and Educational Purposes.

### 2.3 Sepsis induction

Sepsis was induced via the cecal ligation and puncture (CLP) model, according to the protocol described by Alverdy et al. (11). Briefly, after anesthesia with isoflurane (2–3%), a midline laparotomy was performed and the cecum was exteriorized. The cecum was ligated distally to the ileocecal valve without causing intestinal obstruction. Subsequently, the cecum was punctured five times with a 25-gauge needle (0.7 mm) and gently squeezed to extrude a small amount of fecal matter into the abdominal cavity. The organ was returned to the peritoneal cavity, and the incision was closed with two layers of sutures. Postoperatively, all rats received 1 mL/100 g of 0.9% NaCl (saline) at 37 °C intraperitoneally for hydration. Immediately following the surgical procedure, the animals were randomized into 6 experimental groups of 10 rats each. Animal health was monitored by two investigators every 2 h for the first 12 h post-induction, and twice daily thereafter. Rats were evaluated in their home cages with the lids removed for better visualization. Sepsis was confirmed according to the Murine Sepsis Score (MSS) proposed by Shrum et al. (12), which assesses spontaneous movement, response to tactile and auditory stimuli, posture, respiratory rate and quality, and appearance (i.e., degree of piloerection). All septic animals exhibited a mean MSS of 3, characterized by piloerection, reduced locomotor activity (trembling movements only when provoked), lack of response to auditory stimuli, half-closed eyes with reddish secretions (porphyrin), and moderately reduced, labored (gasping) breathing.

Throughout the experimental period, no analgesics were administered to avoid the risk of these drugs interfering with systemic immune responses and hemodynamic variables, which could mask the pathophysiology of sepsis and compromise the validity of the experimental data; however, this practice was mitigated by the rigorous implementation of humane endpoints. These endpoints consisted of strictly defined early termination clinical criteria designed to prevent unnecessary suffering. These included severe prostration with an inability to respond to external stimuli, persistent or profound hypothermia, evident respiratory distress, and the failure to maintain normal posture or grooming habits. The manifestation of any of these indicators, whether in isolation or combination, necessitated the immediate euthanasia of the animal to ensure that ethical welfare standards took precedence over further data collection.

### 2.4 Anesthetic protocol selection

Five anesthetic protocols for rats were selected for this study: (1) ketamine (100 mg/kg; IP) and xylazine (10 mg/kg; IP) according to Wellington et al. (13); (2) fentanyl (0.5 mg/kg; IP) plus diazepam (2.5 mg/kg; IP) following the methods described by Psatha et al. (14); (3) fentanyl (0.5 mg/kg; IP) plus dexmedetomidine (0.5 mg/kg; IP) as proposed by Bhana et al. (15); (4) ketamine (130 mg/kg; IP) plus dexmedetomidine (0.5 mg/kg; IP) according to the protocol by Callahan et al. (16); and (5) inhaled isoflurane (2–3%) as proposed by Woodward et al. (17).

### 2.5 Experimental design

Seven days post-sepsis confirmation, the number of animals per group was adjusted to seven, corresponding to an overall mortality rate of 30%. Five groups were subjected to the anesthetic protocols described in Section 2.4, while a septic control group remained unanesthetized to serve as an internal control. To ensure physiological consistency, all anesthetized animals underwent a standardized procedure with a mean anesthesia duration of 1.5 h. Throughout the study, body temperature was maintained at 37 ± 0.5 °C using a homeothermic heating system to prevent thermoregulatory-induced hemodynamic bias. Animals were maintained under spontaneous breathing, as no intubation or mechanical ventilation was performed. Respiratory rate (80 ± 15 breaths/min) and peripheral oxygen saturation (SpO₂: 90 ± 5%) were continuously monitored using a MouseOx Plus pulse oximeter (Ugo Basile, Gemonio, Italy) to ensure vital signs remained within physiological ranges and to minimize confounding effects of respiratory depression or hypoxia on hemodynamic homeostasis.

To ensure a deep surgical plane of anesthesia, a multimodal assessment was performed throughout the procedures. The depth of anesthesia was confirmed by the absence of pedal withdrawal, palpebral, and auricular reflexes, alongside a complete state of muscle relaxation. Continuous monitoring of physiological parameters was maintained, specifically targeting a slow, regular respiratory pattern and the maintenance of pink mucous membranes. The adequacy of the anesthetic level was validated by the absence of response to a noxious stimulus (skin pinching) prior to the initiation of any surgical incision. All procedures were conducted in strict accordance with the guidelines established by the National Council for the Control of Animal Experimentation (CONCEA). Immediately upon verification of a stable anesthetic state, the respective experimental protocols were initiated.

#### 2.5.1 Electrocardiography (ECG)

Initially, all anesthetized rats were placed in a supine position, while conscious animals (control group) were restrained in individual acrylic holders. Four electrodes were positioned on the carpal and tarsal bones, and a small amount of conductive gel was applied to each electrode interface to ensure optimal electrical contact. After a 5-min acclimation period, the PR, QT, and QTc intervals, the QRS complex, and the P, Q, R, S, and T waves were recorded using a digital ECG recorder (WinCardio, Micromed, Brasília, Brazil).

#### 2.5.2 Blood pressure and heart rate

Following electrocardiography, all animals received a subcutaneous bolus injection of heparin (30 IU). For the anesthetized group, the left carotid artery was isolated and cannulated with a fluid-filled catheter connected to a calibrated pressure transducer (MLT0699; ADInstruments, Bella Vista, NSW, Australia). Signals were amplified and digitized via a PowerLab data acquisition system (ADInstruments) and subsequently analyzed using LabChart software (version 7.3.8). After a 15-min post-surgical stabilization period, blood pressure was recorded for 15 min, and the reported values represent the mean of the final 5 min of this interval.

In contrast, conscious control animals underwent a 2-week conditioning period prior to the non-invasive measurement of systolic blood pressure (SBP), diastolic blood pressure (DBP), and heart rate (HR) using a tail-cuff plethysmography system (NIBP System; ADInstruments). During these measurements, animals were placed in a restrainer and kept on water-circulating heating pads to ensure adequate peripheral blood flow (tail temperature of 35 °C). In this group, mean arterial pressure (MAP) was calculated as MAP = DBP + 1/3 (SBP − DBP), and reported values represent the mean of three consecutive measurements taken at 15-min intervals. To calculate the coefficient of variation (CV), we use the formula: CV = (standard deviation/mean) × 100.

#### 2.5.3 Blood collection and arterial blood gas analysis

Following blood pressure measurements, blood samples (4–5 mL) were collected from anesthetized animals via a cannula in the left carotid artery. In conscious animals, arterial blood samples were obtained by puncturing the ventral tail artery while the rats were restrained in clear acrylic holders designed for animals weighing 250–300 g (internal dimensions: 60 mm × 200 mm diameter × length; Bonther, Ribeirão Preto, SP, Brazil).

For arterial blood gas analysis, the following parameters were measured using a multiparameter blood gas analyzer (Cobas b 221; Roche Diagnostics, Rotkreuz, Switzerland): pH, pCO_2_ (partial pressure of carbon dioxide), pO_2_ (partial pressure of oxygen), Na^+^ (sodium), K^+^ (potassium), Cl^−^ (chloride), Ca^++^ (ionized calcium), TCO_2_ (total carbon dioxide concentration), glucose, lactate, BUN (blood urea nitrogen), creatinine, Hct (hematocrit), cHgb (hemoglobin), cHCO_3_^−^ (bicarbonate), BE (base excess) (ecf), BE (b), cSO_2_ (oxygen saturation), and Agap (anion gap).

#### 2.5.4 Serum biochemical analysis

For serum biochemical analyses, serum was obtained by centrifugation (1,500 × *g* for 10 min). Serum levels of urea, creatinine, cardiac troponin C, creatine kinase-MB (CK-MB), sodium, and potassium were measured using an automated biochemical analyzer (Roche Cobas Integra 400 Plus). Nitrotyrosine (NT) and aldosterone levels were determined by enzyme-linked immunosorbent assay (ELISA) (MyBioSource, San Diego, CA, USA). Malondialdehyde (MDA) levels were measured using an MDA assay kit (Cayman Chemical, Ann Arbor, MI, USA). Plasma angiotensin-converting enzyme (ACE) activity was determined according to the technique described by Santos et al. (18).

#### 2.5.5 Mesenteric vascular reactivity

Immediately after blood collection, the mesenteric vascular beds (MVBs) were isolated from anesthetized animals, cannulated, and prepared according to the methods previously described by Kawasaki et al. (19). The MVBs were continuously perfused with physiological salt solution (PSS) containing 119 mM NaCl, 4.7 mM KCl, 2.4 mM CaCl_2_, 1.2 mM MgSO_4_, 25.0 mM NaHCO_3_, 1.2 mM KH2PO4, 11.1 mM dextrose, and 0.03 mM EDTA The solution was oxygenated with a gas mixture of 95% O_2_ and 5% CO_2_ and maintained at 37 °C. Vascular reactivity was measured using a Radnoti system (ADInstruments, Bella Vista, NSW, Australia). High-precision, water-jacketed borosilicate glass chambers were employed to ensure rigorous thermal control, which is essential for maintaining tissue stability. A constant flow rate of 4 mL/min was maintained using an electronic peristaltic perfusion pump (Samtronic, São Paulo, SP, Brazil). Data were acquired using a PowerLab data acquisition system and analyzed via LabChart software (version 8; ADInstruments, Bella Vista, NSW, Australia).

Following a 30-min stabilization period, tissue integrity was verified via a bolus injection of 120 mM KCl. Subsequently, phenylephrine (Phe; 1, 3, 10, 30, and 100 nmol; 10–30 μL) was administered as individual doses. After another 30-min stabilization period, the MVBs were continuously perfused with PSS containing 3 μM Phe. Once the contractile response reached a plateau, acetylcholine (ACh; 0.01, 0.03, 0.1, 0.3, and 1 nmol; 10–30 μL) and sodium nitroprusside (SNP; 0.3, 1, 3, 10, and 30 nmol; 10–30 μL) were administered. Individual drug doses were administered, rather than cumulative concentration-response curves, to ensure a precise characterization of the vascular response to each specific dose. Subsequent doses were administered only after the perfusion pressure returned to pre-administration baseline values. Following the experimental procedures, anesthetized animals were euthanized via a supra-pharmacological dose of the anesthetic combination. Non-anesthetized animals were euthanized by decapitation, followed by the immediate excision of the MVBs.

#### 2.5.6 Histopathological analysis

Following euthanasia, the heart, left kidney, and aorta were removed, longitudinally sectioned, and cleaned. Samples from each organ were fixed in 10% buffered formalin. The specimens were then dehydrated in graded alcohol, cleared in xylene, and embedded in paraffin. Subsequently, 5 μm sections were obtained, stained with hematoxylin and eosin (H&E), and examined under a Nikon ECLIPSE Si binocular optical microscope. All images were captured and analyzed using Motic Images Plus 2.0 software.

### 2.6 Statistical analysis

Data were analyzed for homogeneity of variance and normality. Differences between means were determined using one-way analysis of variance (ANOVA) followed by Bonferroni’s *post hoc* test. Results were expressed as mean ± standard error of the mean (SEM), and *p*-values <0.05 were considered statistically significant. Statistical analyses were performed using GraphPad Prism software version 11 for macOS (GraphPad® Software, San Diego, CA, USA).

## 3 Results

### 3.1 Electrocardiographic changes

The electrocardiographic parameters for septic SHR rats anesthetized with different pharmacological protocols are summarized in Table 1. All septic SHR animals anesthetized with alpha 2-adrenergic agonists (ketamine/xylazine, fentanyl/dexmedetomidine, and ketamine/dexmedetomidine) exhibited significant prolongation of the QRS complex and QT interval, alongside a reduction in R-wave amplitude compared to the control, isoflurane, and fentanyl/diazepam groups. Conversely, rats anesthetized with either isoflurane or the fentanyl/diazepam combination maintained electrocardiographic values similar to those of non-anesthetized animals (control group).

**Table 1:** Electrocardiographic data of septic spontaneously hypertensive rats (SHR) anesthetized with different pharmacological protocols.

Parameters	Control	Isoflurane (2–3%; INH)	Ketamine plus xylazine (100/10 mg/kg; IP)	Fentanyl plus diazepam (0.5/2.5 mg/kg; IP)	Fentanyl plus dexmedetomidine (0.5/0.5 mg/kg; IP)	Ketamine plus dexmedetomidine (130/0.5 mg/kg; IP)
PR interval (ms)	43.11 ± 4.13	41.14 ± 2.77	46.43 ± 3.84	47.29 ± 2.52	50.00 ± 4.05	48.50 ± 3.88
QRS complex (ms)	44.35 ± 4.21	40.29 ± 4.94	**75.63 ± 4.86** ^**abc**^	39.17 ± 4.15	**79.22 ± 4.81** ^**abc**^	**71.00 ± 4.23** ^**abc**^
QT interval (ms)	83.12 ± 4.11	85.86 ± 5.02	**112.9 ± 6.02** ^**abc**^	81.1 ± 5.04	**115.43 ± 7.95** ^**abc**^	**119.6 ± 8.83** ^**abc**^
QTc interval (ms)	219.2 ± 13.21	212.1 ± 14.98	220.3 ± 10.92	214.8 ± 14.88	213.9 ± 9.29	211.8 ± 9.88
P wave (mV)	0.03 ± 0.01	0.04 ± 0.01	0.04 ± 0.01	0.05 ± 0.02	0.05 ± 0.02	0.04 ± 0.01
Q wave (mV)	−0.02 ± 0.01	−0.02 ± 0.01	−0.02 ± 0.01	−0.02 ± 0.01	−0.02 ± 0.01	−0.02 ± 0.01
R wave (mV)	0.22 ± 0.04	0.24 ± 0.04	**0.15 ± 0.02** ^**abc**^	0.22 ± 0.02	**0.13 ± 0.02** ^**abc**^	**0.14 ± 0.02** ^**abc**^
S wave (mV)	−0.02 ± 0.01	−0.02 ± 0.01	−0.03 ± 0.01	−0.03 ± 0.01	−0.03 ± 0.01	−0.03 ± 0.01
T wave (mV)	0.05 ± 0.02	0.04 ± 0.01	0.05 ± 0.02	0.04 ± 0.01	0.05 ± 0.02	0.04 ± 0.01

Statistical analyses were performed using one-way ANOVA followed by Bonferroni’s *post hoc* test. Data are expressed as mean ± standard error of the mean (SEM) (*n* = 7). ^a^*p* < 0.05 vs. control group; ^b^*p* < 0.05 vs. isoflurane group; ^c^*p* < 0.05 vs. fentanyl + diazepam group. INH, inhalation route; IP, intraperitoneal route. Bold values (mean ± SEM) indicate statistically significant differences.

### 3.2 Hemodynamic effects

Blood pressure and heart rate (HR) values of septic SHR anesthetized with different pharmacological protocols are presented in Table 2. Septic animals anesthetized with isoflurane exhibited mean SBP, DBP, MAP, and HR values similar to those of non-anesthetized septic rats measured by the indirect tail-cuff method. In contrast, all animals anesthetized with ketamine/xylazine, fentanyl/diazepam, fentanyl/dexmedetomidine, or ketamine/dexmedetomidine showed significantly lower SBP, DBP, and MAP values compared to the control and isoflurane groups. Regarding heart rate, rats anesthetized with fentanyl/diazepam maintained values similar to the control and isoflurane groups, whereas these were significantly lower in the groups anesthetized with ketamine/xylazine, fentanyl/dexmedetomidine, and ketamine/dexmedetomidine. It is observed that the groups anesthetized with injectable combinations (particularly those with dexmedetomidine and fentanyl/diazepam) generally exhibit higher CVs compared to the control and isoflurane groups (Table 3). The control and isoflurane groups maintained lower coefficients of variation across most hemodynamic parameters, reinforcing the interpretation that isoflurane better preserves hemodynamic stability in the studied sepsis model.

**Table 2:** Blood pressure and heart rate of septic spontaneously hypertensive rats (SHR) anesthetized with different pharmacological protocols.

Parameters	Control	Isoflurane (2–3%; INH)	Ketamine plus xylazine (100/10 mg/kg; IP)	Fentanyl plus diazepam (0.5/2.5 mg/kg; IP)	Fentanyl plus dexmedetomidine (0.5/0.5 mg/kg; IP)	Ketamine plus dexmedetomidine (130/0.5 mg/kg; IP)
SBP (mm Hg)	169.1 ± 8.83	165.6 ± 9.37	**111.5 ± 7.57** ^**ab**^	**121.3 ± 7.68** ^**ab**^	**100.4 ± 10.40** ^**ab**^	**92.97 ± 9.24** ^**ab**^
DBP (mm Hg)	95.12 ± 7.47	97.44 ± 8.65	**57.19 ± 6.55** ^**ab**^	**55.42 ± 11.50** ^**ab**^	**32.31 ± 4.80** ^**ab**^	**43.67 ± 9.35** ^**ab**^
MAP (mm Hg)	123.2 ± 6.66	125.7 ± 9.37	**82.27 ± 6.51** ^**ab**^	**88.21 ± 11.30** ^**ab**^	**57.04 ± 5.30** ^**ab**^	**66.27 ± 8.12** ^**ab**^
HR (bpm)	355.2 ± 24.13	345.8 ± 31.05	**151.3 ± 22.37** ^**abc**^	299.0 ± 33.72	**208.4 ± 25.62** ^**abc**^	**171.4 ± 30.83** ^**abc**^

Statistical analyses were performed using one-way ANOVA followed by Bonferroni’s *post hoc* test. Data are expressed as mean ± standard error of the mean (SEM) (*n* = 7). ^a^*p* < 0.05 vs. control group; ^b^*p* < 0.05 vs. isoflurane group; ^c^*p* < 0.05 vs. fentanyl + diazepam group. DBP, diastolic blood pressure; HR, heart rate; INH, inhalation route; IP, intraperitoneal route; MAP, mean arterial pressure; SBP, systolic blood pressure. Bold values (mean ± SEM) indicate statistically significant differences.

**Table 3:** Coefficient of variation (%) for blood pressure and heart rate values of septic spontaneously hypertensive rats (SHR) anesthetized with different pharmacological protocols.

Parameters	Control	Isoflurane (2–3%; INH)	Ketamine plus xylazine (100/10 mg/kg; IP)	Fentanyl plus diazepam (0.5/2.5 mg/kg; IP)	Fentanyl plus dexmedetomidine (0.5/0.5 mg/kg; IP)	Ketamine plus dexmedetomidine (130/0.5 mg/kg; IP)
SBP (%)	13.8	14.9	17.9	15.8	26.0	26.3
DBP (%)	20.7	23.4	30.2	55.0	39.2	56.7
MAP (%)	13.9	19.7	20.9	33.8	24.5	32.4
HR (%)	17.9	23.7	39.0	29.8	32.5	47.5

Statistical analyses were performed using one-way ANOVA followed by Bonferroni’s *post hoc* test. The coefficient of variation (CV) was calculated using the formula: CV = (standard deviation/mean) × 100. DBP, diastolic blood pressure; HR, heart rate; INH, inhalation route; IP, intraperitoneal route; MAP, mean arterial pressure; SBP, systolic blood pressure. Bold values (mean ± SEM) indicate statistically significant differences.

### 3.3 Arterial blood gas

Arterial blood gas parameters of septic spontaneously hypertensive rats (SHR) under different anesthetic protocols are presented in Table 4. Septic animals anesthetized with isoflurane exhibited a gasometric profile similar to the control group (septic unanesthetized SHR). In contrast, septic SHR treated with ketamine/xylazine, fentanyl/diazepam, fentanyl/dexmedetomidine, and ketamine/dexmedetomidine showed significantly higher PCO_2_ and Ca^2+^ levels, alongside significantly lower pH and PO_2_ values compared to both the isoflurane and unanesthetized groups. No other parameters were significantly affected by the anesthetic protocols.

**Table 4:** Arterial blood gas and biochemical profile of septic spontaneously hypertensive rats (SHR) subjected to different anesthetic protocols.

Parameters	Control	Isoflurane (2–3%; INH)	Ketamine plus xylazine (100/10 mg/kg; IP)	Fentanyl plus diazepam (0.5/2.5 mg/kg; IP)	Fentanyl plus dexmedetomidine (0.5/0.5 mg/kg; IP)	Ketamine plus dexmedetomidine (130/0.5 mg/kg; IP)
pH	7.35 ± 0.04	7.37 ± 0.03	**7.07 ± 0.08** ^**ab**^	**7.03 ± 0.07** ^**ab**^	**6.89 ± 0.09** ^**ab**^	**6.93 ± 0.05** ^**ab**^
pCO_2_ (mmHg)	35.77 ± 5.12	38.92 ± 4.09	**93.50 ± 17.73** ^**ab**^	**93.28 ± 12.77** ^**ab**^	**99.86 ± 6.03** ^**ab**^	**109.3 ± 6.38** ^**ab**^
pO_2_ (mmHg)	121.1 ± 11.43	124.3 ± 13.39	**90.18 ± 9.17** ^**ab**^	**94.80 ± 8.66** ^**ab**^	**91.84 ± 13.20** ^**ab**^	**98.80 ± 7.32** ^**ab**^
Na^+^ (mEq/L)	123.7 ± 4.55	126.4 ± 4.35	131.2 ± 4.33	139.4 ± 4.77	135.6 ± 3.58	136.0 ± 5.81
K^+^ (mEq/L)	5.17 ± 0.41	5.28 ± 0.48	6.52 ± 0.92	4.68 ± 0.33	7.72 ± 1.53	6.54 ± 0.81
Cl^−^ (mEq/L)	95.43 ± 2.78	96.00 ± 2.68	104.20 ± 2.93	104.1 ± 2.07	103.4 ± 2.22	104.8 ± 2.98
Ca^2+^ (mmol/L)	1.19 ± 0.03	1.17 ± 0.03	**1.32 ± 0.02** ^**ab**^	**1.30 ± 0.03** ^**ab**^	**1.43 ± 0.04** ^**ab**^	**1.45 ± 0.05** ^**ab**^
TCO_2_ (mmol/L)	21.15 ± 1.12	22.16 ± 2.70	27.28 ± 3.82	25.50 ± 1.24	22.42 ± 3.56	25.50 ± 2.03
Glucose (mg/dL)	355.8 ± 33.77	315.6 ± 36.93	345.0 ± 31.55	324.8 ± 37.02	338.0 ± 31.22	345.8 ± 38.75
Lactate (mmol/L)	4.26 ± 0.43	4.22 ± 0.51	6.94 ± 2.78	6.60 ± 0.89	10.22 ± 3.40	7.91 ± 1.31
BUN (mg/dL)	33.41 ± 1.55	30.40 ± 1.74	37.00 ± 4.91	36.40 ± 2.11	35.60 ± 2.29	31.40 ± 1.63
Creatinine (mg/dL)	0.39 ± 0.23	0.33 ± 0.21	0.56 ± 0.11	0.57 ± 0.04	0.36 ± 0.04	0.31 ± 0.00
Hct (%)	42.12 ± 2.66	41.00 ± 2.54	35.80 ± 4.07	39.20 ± 1.96	44.00 ± 3.46	39.40 ± 1.16
cHgb (g/dL)	13.65 ± 0.55	13.88 ± 0.22	12.20 ± 1.39	13.34 ± 0.63	14.90 ± 1.19	13.40 ± 0.36
cHCO_3_^−^ (mEq/L)	21.86 ± 0.89	22.66 ± 0.61	25.80 ± 1.45	23.96 ± 1.19	20.54 ± 3.67	23.64 ± 2.12
BE (ecf) (mEq/L)	−2.37 ± 0.83	−2.46 ± 0.87	−4.14 ± 1.76	−3.72 ± 1.01	−4.40 ± 2.26	−3.62 ± 1.02
BE (b) (mmol/L)	−2.24 ± 0.31	−2.14 ± 0.39	−2.42 ± 0.71	−2.34 ± 0.79	−2.92 ± 0.48	−2.00 ± 0.94
cSO_2_ (%)	66.99 ± 11.22	61.74 ± 12.82	79.92 ± 11.00	77.06 ± 19.57	62.96 ± 10.12	53.06 ± 10.33
Agap (mEq/L)	11.77 ± 2.87	9.20 ± 1.02	12.00 ± 3.61	9.88 ± 1.02	9.80 ± 1.77	11.80 ± 2.39

Statistical analyses were performed using one-way ANOVA followed by Bonferroni’s *post hoc* test. Data are expressed as mean ± standard error of the mean (SEM) (*n* = 7). ^a^*p* < 0.05 vs. control group; ^b^*p* < 0.05 vs. isoflurane group. pH, potential of hydrogen; PCO_2_, partial pressure of carbon dioxide; PO_2_, partial pressure of oxygen; Na^+^, sodium; K^+^, potassium; Cl^−^, chloride; Ca^2+^, calcium; TCO_2_, total carbon dioxide; BUN, blood urea nitrogen; Hct, hematocrit; CHgb, hemoglobin concentration; HCO_3_^−^, bicarbonate; BE, base excess; O_2_, oxygen saturation; Agap, anion gap; INH, inhalation route; IP, intraperitoneal route. Bold values (mean ± SEM) indicate statistically significant differences.

### 3.4 Serum biochemical parameters

Serum levels of various biochemical parameters in septic SHR anesthetized with different pharmacological agents are presented in Table 5. Septic SHR anesthetized with ketamine/xylazine, fentanyl/diazepam, fentanyl/dexmedetomidine, and ketamine/dexmedetomidine showed significantly higher MDA and serum creatinine levels compared to both the isoflurane and control groups. Furthermore, biochemical parameters in the isoflurane group were statistically similar to those observed in the control group (unanesthetized septic animals).

**Table 5:** Serum biochemical parameters of septic spontaneously hypertensive rats (SHR) anesthetized with different pharmacological protocols.

Parameters	Control	Isoflurane (2–3%; INH)	Ketamine plus xylazine (100/10 mg/kg; IP)	Fentanyl plus diazepam (0.5/2.5 mg/kg; IP)	Fentanyl plus dexmedetomidine (0.5/0.5 mg/kg; IP)	Ketamine plus dexmedetomidine (130/0.5 mg/kg; IP)
NT (μmol/L)	0.021 ± 0.003	0.022 ± 0.004	0.020 ± 0.002	0.021 ± 0.003	0.023 ± 0.004	0.022 ± 0.003
MDA (mmol/L)	2.12 ± 0.24	2.20 ± 0.26	**3.10 ± 0.33** ^**ab**^	**2.99 ± 0.29** ^**ab**^	**3.07 ± 0.31** ^**ab**^	**3.05 ± 0.31** ^**ab**^
Urea (mg/dL)	33.1 ± 5.21	31.6 ± 3.75	37.4 ± 4.21	30.2 ± 4.42	33.9 ± 5.12	30.6 ± 4.12
Creatinine (mg/dL)	1.33 ± 0.21	1.29 ± 0.24	**1.91 ± 0.21** ^**ab**^	**1.91 ± 0.18** ^**ab**^	**1.88 ± 0.17** ^**ab**^	**1.94 ± 0.19** ^**ab**^
Sodium (mmol/L)	125.1 ± 3.5	129.3 ± 5.6	127.2 ± 5.1	121.2 ± 4.9	124.7 ± 6.6	127.2 ± 5.2
Potassium (mmol/L)	5.22 ± 0.52	5.34 ± 0.44	5.53 ± 0.71	5.37 ± 0.63	5.52 ± 0.55	5.45 ± 0.73
Aldosterone (pg/mL)	171 ± 11.21	172 ± 13.2	161 ± 12.7	177 ± 12.2	178 ± 15.5	180 ± 15.5
ACE activity (mmol/min/mL)	110 ± 11.21	114 ± 13.32	117 ± 14.77	116 ± 15.21	111 ± 14.21	115 ± 12.11
CK-MB (U/L)	89.11 ± 15.77	92.32 ± 14.13	84.12 ± 17.11	80.12 ± 12.77	82.21 ± 11.33	90.6 ± 14.55
TnC (μg/L)	0.15 ± 0.03	0.17 ± 0.03	0.16 ± 0.03	0.17 ± 0.03	0.18 ± 0.03	0.16 ± 0.03

Statistical analyses were performed using one-way ANOVA followed by Bonferroni’s *post hoc* test. Data are expressed as mean ± standard error of the mean (SEM) (*n* = 7). ^a^*p* < 0.05 vs. control group; ^b^*p* < 0.05 vs. isoflurane group. ACE, angiotensin-converting enzyme; CK-MB, creatine phosphokinase MB fraction; TnC, cardiac troponin C; MDA, malondialdehyde; NT, nitrotyrosine; INH, inhalation route; IP, intraperitoneal route. Bold values (mean ± SEM) indicate statistically significant differences.

### 3.5 Effects on mesenteric vascular reactivity

Mesenteric vascular reactivity values of septic SHR anesthetized with different pharmacological protocols are presented in Table 6. Septic SHR anesthetized with ketamine/xylazine, fentanyl/diazepam, fentanyl/dexmedetomidine, and ketamine/dexmedetomidine showed a significantly reduced vasodilatory response to ACh (0.1, 0.3, and 1 nmol) when compared to control animals or those anesthetized with isoflurane. The vasodilatory response to ACh in isoflurane-anesthetized animals was statistically similar to that observed in the control group (non-anesthetized septic rats). Vascular responses to Phe and SNP were statistically similar across all experimental groups.

**Table 6:** Mesenteric vascular reactivity (*Δ* values; mm Hg*) of septic spontaneously hypertensive rats (SHR) anesthetized with different pharmacological protocols.

Parameters	Control	Isoflurane (2–3%; INH)	Ketamine plus xylazine (100/10 mg/kg; IP)	Fentanyl plus diazepam (0.5/2.5 mg/kg; IP)	Fentanyl plus dexmedetomidine (0.5/0.5 mg/kg; IP)	Ketamine plus dexmedetomidine (130/0.5 mg/kg; IP)
Phe (nmol)						
1	10.13 ± 0.56	13.87 ± 3.88	13.01 ± 3.27	12.63 ± 2.91	12.21 ± 2.54	12.73 ± 2.89
3	20.12 ± 2.33	17.99 ± 2.25	15.66 ± 3.83	18.66 ± 2.19	18.96 ± 2.18	17.14 ± 2.52
10	48.11 ± 9.21	42.79 ± 4.44	46.30 ± 4.56	44.99 ± 4.08	44.22 ± 4.37	45.78 ± 4.61
30	90.11 ± 9.13	93.02 ± 11.71	93.65 ± 11.14	98.68 ± 9.36	93.47 ± 0.7.75	95.26 ± 7.94
100	120.30 ± 13.12	125.32 ± 17.99	124.45 ± 13.65	123.39 ± 17.01	125.12 ± 15.93	129.74 ± 10.99
ACh (nmol)						
0.01	−4.78 ± 0.88	−4.01 ± 0.67	−3.76 ± 1.63	−3.99 ± 1.17	−5.58 ± 2.19	−5.79 ± 1.84
0.03	−9.21 ± 2.66	−11.21 ± 1.47	−8.28 ± 3.04	−9.41 ± 2.96	−10.60 ± 1.88	−11.97 ± 3.81
0.1	−25.12 ± 4.12	−27.69 ± 6.12	**−13.68 ± 3.12** ^**ab**^	**−12.05 ± 2.18** ^**ab**^	**−12.07 ± 3.25** ^**ab**^	**−12.82 ± 2.94** ^**ab**^
0.3	−34.33 ± 6.24	−33.99 ± 5.73	**−17.23 ± 3.95** ^**ab**^	**−14.69 ± 2.37** ^**ab**^	**−16.41 ± 3.83** ^**ab**^	**−15.44 ± 2.14** ^**ab**^
1	−36.42 ± 4.12	−33.81 ± 7.49	**−16.53 ± 6.55** ^**ab**^	**−16.83 ± 4.44** ^**ab**^	**−18.93 ± 4.22** ^**ab**^	**−20.34 ± 3.10** ^**ab**^
NPS (nmol)						
0.3	−5.21 ± 1.57	−5.39 ± 1.04	−8.20 ± 3.23	−5.93 ± 1.49	−7.04 ± 3.46	−8.27 ± 4.20
1	−7.77 ± 2.12	−7.85 ± 2.42	−10.28 ± 5.72	−10.71 ± 2.60	−11.99 ± 4.86	−10.46 ± 4.73
3	−25.14 ± 6.44	−24.25 ± 8.48	−26.79 ± 5.92	−25.39 ± 2.76	−19.62 ± 5.78	−22.20 ± 7.48
10	−31.44 ± 5.34	−30.31 ± 9.07	−37.02 ± 5.23	−39.38 ± 5.38	−38.57 ± 2.70	−35.86 ± 9.16
30	−36.25 ± 3.45	−35.13 ± 10.18	−37.83 ± 3.09	−32.67 ± 3.09	−38.47 ± 4.13	−34.85 ± 8.31

*Data are expressed as changes (mm Hg) from baseline (*Δ* values). Each dose was administered individually; therefore, values represent the difference between the post-administration stabilization plateau and the specific baseline recorded immediately prior to each dose. Statistical analyses were performed using one-way ANOVA followed by Bonferroni’s *post hoc* test. Data are expressed as mean ± standard error of the mean (SEM) (*n* = 7). ^a^*p* < 0.05 vs. control group; ^b^*p* < 0.05 vs. isoflurane group. ACh, acetylcholine; Phe, phenylephrine; SNP, sodium nitroprusside; INH, inhalation route; IP, intraperitoneal route. Bold values (mean ± SEM) indicate statistically significant differences.

### 3.6 Histopathological analysis

Representative histological images of the left ventricle, left kidney, and aorta from septic SHR anesthetized with different pharmacological protocols are shown in Supplementary Figures 1–3. None of the experimental groups showed signs of cellular infiltration, inflammation, fibrosis, hypoplasia, hyperplasia, hypertrophy, ischemia, apoptosis, or necrosis in the evaluated tissues.

## 4 Discussion

The maintenance of hemodynamic stability is a cornerstone of preclinical drug discovery, particularly when evaluating novel compounds with cardiovascular activity. While direct blood pressure measurement via carotid artery cannulation remains the gold standard for high-fidelity monitoring in rodent models (20, 21), the physiological relevance of these parameters is intrinsically linked to the anesthetic regimen employed. Selecting an appropriate protocol is a critical methodological variable, as many traditional agents induce autonomic depression, alter baroreflex sensitivity, or exert negative inotropic effects (22, 23).

The primary objective of this study was to identify an anesthetic protocol that minimizes interference with sepsis-related hemodynamic alterations, thereby reducing experimental bias and ensuring a reliable representation of the disease state. To model sepsis, we employed the cecal ligation and puncture (CLP) method. Despite its invasive nature, CLP remains the gold standard for polymicrobial sepsis research because it faithfully replicates the pathophysiological progression, immunological complexity, and hemodynamic responses observed in clinical settings (24). While alternative approaches—such as endotoxin administration or fecal-induced peritonitis—offer higher reproducibility and reduced surgical trauma, CLP superiorly preserves a continuous source of infection and simulates intestinal barrier failure and bacterial translocation. These attributes render it crucial for translating experimental findings into clinical practice (25).

However, outcomes in the CLP model can vary significantly depending on the needle gauge, number of punctures, extent of cecal ligation, fluid resuscitation, and antibiotic administration. In moderate sepsis models, mortality typically ranges between 30 and 50% over a 7- to 14-day observation window, offering an ideal therapeutic timeframe to investigate potential interventions and host survival mechanisms (26, 27). Aligning with these benchmarks, our study demonstrated a 30% mortality rate—accounting strictly for spontaneous deaths in the absence of antibiotic treatment or early euthanasia based on humane endpoints.

Our findings demonstrate that anesthetic combinations incorporating alpha-adrenergic agonists—specifically ketamine-xylazine, fentanyl-dexmedetomidine, and ketamine-dexmedetomidine—are unsuitable for hemodynamic studies in septic rats. These regimens failed to maintain stability, likely due to their significant sympatholytic and cardiovascular-depressant effects, which exacerbate the cardiovascular dysfunction already present in the model. Consistent with previous reports (28, 29), we observed significant reductions in blood pressure and impaired autonomic modulation with ketamine-xylazine. While others have suggested dexmedetomidine may offer cardioprotection against ischemia–reperfusion (30), our data indicate that in a septic context, its associated bradycardia and hypotension are detrimental, likely driven by alpha-2 adrenoceptor activation in the locus coeruleus and subsequent K+ channel-mediated hyperpolarization (31–33).

Furthermore, our study highlights the risks of incorporating opioids like fentanyl into septic models. We observed that fentanyl, when combined with diazepam or dexmedetomidine, induced significant hemodynamic depression, tissue hypoxia, oxidative damage, and renal impairment. Mechanistically, this is supported by fentanyl’s modulation of μ-opioid receptors, which leads to vagal stimulation, reduced sympathetic tone, and inhibition of vascular contraction pathways (34–36). While fentanyl provides effective analgesia, our results indicate that, in compromised septic models, it significantly aggravates vascular collapse and cardiovascular instability, echoing patterns seen in severe physiological distress (37).

Our findings indicate that isoflurane is the adequate anesthetic for this model, as it preserves hemodynamic, gasometric, and biochemical parameters at levels comparable to unanesthetized controls, thereby minimizing interference with the septic phenotype. Unlike alpha-2 agonists or opioids, which often cause autonomic disruption and negative inotropic effects (30, 31), isoflurane maintains the vascular response to ACh, allowing for a clear observation of the disease state. Furthermore, we recognize that while isoflurane can provide cardioprotection via PI3K/Akt and KATP channel pathways—effects that are strictly dose- and duration-dependent—it must be used cautiously to avoid paradoxically impairing immune function and hemodynamic stability in septic subjects (38–41). By employing a strictly controlled, minimal-dose protocol, we successfully mitigated these potential confounding factors, including anesthetic-induced pre-conditioning and cardiovascular depression, ensuring that our data accurately reflect the underlying CLP-induced pathophysiology rather than anesthetic modulation.

It is important to acknowledge that the comparative assessment between isoflurane-anesthetized animals and unanesthetized controls involved different measurement modalities—direct carotid cannulation versus non-invasive tail-cuff plethysmography, respectively. While these techniques are standard, they are not directly equivalent, which may introduce a methodological bias. However, the superior stability observed with isoflurane, coupled with its ability to maintain physiological responses during hypoxic or hypotensive insults (42), suggests it is the most appropriate choice for maintaining the integrity of the septic model. Future research employing invasive monitoring across all groups, including unanesthetized baseline controls, will be essential to further validate these findings and fully isolate the impact of anesthesia from the underlying disease progression.

A limitation of this study is the lack of evaluation of propofol, one of the most widely used intravenous anesthetics in clinical and experimental settings due to its rapid onset, smooth recovery, and titratable depth of sedation (43). Although propofol provides distinct cardiovascular and pharmacological advantages—such as predictable anesthetic control and rapid clearance—incorporating it into our experimental design presented significant methodological constraints. Intravenous administration of propofol in conscious rats typically requires invasive vascular cannulation under severe physical restraint or premedication. Baseline sedation would introduce a drug-combination effect, confounding the direct cardiovascular impact of the anesthetic agent on the septic phenotype. Conversely, physical restraint without sedation would trigger severe neuroendocrine and hemodynamic stress responses, significantly distorting baseline septic parameters. Therefore, to prevent these methodological biases and ensure an isolated evaluation of each anesthetic protocol in spontaneously hypertensive rats subjected to CLP-induced hemodynamic instability, propofol was excluded. Future investigations employing catheterized animal models are recommended to evaluate its hemodynamic safety in this context.

## 5 Conclusion

In conclusion, while isoflurane demonstrated a favorable hemodynamic profile in spontaneously hypertensive rats subjected to sepsis-induced instability compared to the evaluated injectable protocols, characterizing it as definitively “superior” may be an overinterpretation without broader comparative data, such as non-septic control groups or long-term survival outcomes. Our findings, therefore, indicate that isoflurane provides optimal stability specifically within the constraints of this experimental model, emphasizing that anesthetic protocol selection must be rigorously tailored to the cardiovascular vulnerabilities of the septic subjects under investigation.

## Data Availability

The raw data supporting the conclusions of this article will be made available by the authors, without undue reservation.
