# Supplementary material for: Comparative analysis of anesthetic protocols in spontaneously hypertensive rats with sepsis-induced hemodynamic instability
Source: Front Vet Sci. 2026 Aug 19;13:1866908. doi: 10.3389/fvets.2026.1866908 (PMC13533722; doi:10.3389/fvets.2026.1866908)
Supplement: Supplementary file 1 [file Data_Sheet_1.docx]

Supplementary Material

# 1 Supplementary figures

|  |  |  |
| --- | --- | --- |
| Control; Heart, HE 40X | Isoflurane; Heart, HE 40X | Ketamine/xylazine; Heart, HE 40X |
|  |  |  |
| Fentanyl/diazepam; Heart, HE 40X | Fentanyl/Dexmedetomidine; Heart, HE 40X | Ketamine/Dexmedetomidine; Heart, HE 40X |

**Supplementary Figure 1.** Representative histopathological sections of the heart from septic spontaneously hypertensive rats (SHR) anesthetized with different pharmacological protocols. All samples were within normal limits for the species and sex. H&E: hematoxylin and eosin stain.

|  |  |  |
| --- | --- | --- |
| Control; Kidney, HE 40X | Isoflurane; Kidney, HE 40X | Ketamine/xylazine; Kidney, HE 40X |
|  |  |  |
| Fentanyl/Diazepam; Kidney, HE 40X | Fentanyl/Dexmedetomidine; Kidney, HE 40X | Ketamine/Dexmedetomidine; Kidney, HE 40X |

**Supplementary Figure 2.** Representative histopathological sections of the left kidney from septic spontaneously hypertensive rats (SHR) anesthetized with different pharmacological protocols. All samples were within normal limits for the species and sex. H&E: hematoxylin and eosin stain.

|  |  |  |
| --- | --- | --- |
| Control; Aorta, HE 40X | Isoflurane; Aorta, HE 40X | Ketamine/xylazine; Aorta, HE 40X |
|  |  |  |
| Fentanyl/Diazepam; Aorta, HE 40X | Fentanyl/Dexmedetomidine; Aorta, HE 40X | Ketamine/Dexmedetomidine; Aorta, HE 40X |

**Supplementary Figure 3.** Representative histopathological sections of the aorta artery from septic spontaneously hypertensive rats (SHR) anesthetized with different pharmacological protocols. All samples were within normal limits for the species and sex. H&E: hematoxylin and eosin stain.
